# Proceedings of the Brooklyn Dental Society

**Published:** 1871-12

**Authors:** John C. Wyman


### PROCEEDINGS OF THE BROOKLYN DENTAL SOCIETY.

The Brooklyn Dental Society held its regular annual meeting, at the office of Dr. G. A. Mills, on Monday evening, October 2d, and the following gentlemen were elected as officers for the ensuing year:

*President*—Dr. A. H. Brockway.
*Vice President*—Dr. Wm. Jarvis, Jr.
*Recording Secretary*—Dr. John C. Wyman.
*Corresponding Secretary*—Dr. C. A. Marvin.
*Treasurer*—Dr. J. C. Monroe.
*Librarian*—Dr. O. W. Hill.

The attendance was large, and an excellent interest sustained through the whole session.

After the election, the retiring President, Dr. G. A. Mills, made a happy address, which was followed by an appropriate and neatly worded speech from the President-elect.

Prof. Taft, of Cincinnati, also honored the society by his presence, and being invited to speak, he made a telling speech in favor of increased earnestness on the part of each, in seeking to advance the standard of professional education, and thoroughness in every department of our beloved profession.

As usual, a collation was given at the close of business, and thus was ended one of the most pleasant yearly gatherings of this society.

JOHN C. WYMAN, *Reporter.*

